# Understanding the disease and economic impact of avirulent avian paramyxovirus type 1 (APMV-1) infection in Great Britain

**DOI:** 10.1017/S0950268823001255

**Published:** 2023-08-25

**Authors:** Scott M. Reid, Paul Skinner, David Sutton, Craig S. Ross, Karolina Drewek, Natalia Weremczuk, Ashley C. Banyard, Sahar Mahmood, Karen L Mansfield, Jo Mayers, Saumya S. Thomas, Sharon M. Brookes, Ian H. Brown

**Affiliations:** 1 Department of Virology, Animal and Plant Health Agency, New Haw, UK; 2 Qiagen, Manchester, UK; 3 WOAH/FAO International Reference Laboratory for Avian Influenza, Swine Influenza and Newcastle Disease, Animal and Plant Health Agency, New Haw, UK; 4 APHA Veterinary Investigation Centre Starcross, Exeter, UK

**Keywords:** avirulent avian paramyxovirus type 1, avirulent avian paramyxovirus type 1 infection in Great Britain, Newcastle disease, Newcastle disease vaccination, testing to exclude scheme

## Abstract

Newcastle disease (ND) is a notifiable disease affecting chickens and other avian species caused by virulent strains of *Avian paramyxovirus* type 1 (APMV-1). While outbreaks of ND can have devastating consequences, avirulent strains of APMV-1 generally cause subclinical infections or mild disease. However, viruses can cause different levels of disease in different species and virulence can evolve following cross-species transmission events. This report describes the detection of three cases of avirulent APMV-1 infection in Great Britain (GB). Case 1 emerged from the ‘testing to exclude’ scheme in chickens in Shropshire while cases 2 and 3 were made directly from notifiable avian disease investigations in chicken broilers in Herefordshire and on premises in Wiltshire containing ducks and mixed species, respectively). Class II/genotype I.1.1 APMV-1 from case 1 shared 99.94% identity to the Queensland V4 strain of APMV-1. Class II/genotype II APMV-1 was detected from case 2 while the class II/genotype I.2 virus from case 3 aligned closely with strains isolated from *Anseriformes.* Exclusion of ND through rapid detection of avirulent APMV-1 is important where clinical signs caused by avirulent or virulent APMV-1s could be ambiguous. Understanding the diversity of APMV-1s circulating in GB is critical to understanding disease threat from these adaptable viruses.

## Introduction

Newcastle disease (ND) is a notifiable avian disease (NAD) that affects chickens and other avian species, caused by virulent strains of *Avian paramyxovirus* type 1 (APMV-1) [1], also referred to as Newcastle disease virus (NDV). The disease is a serious threat to poultry rearing on a global scale [2]. Outbreaks can have devastating consequences, including up to 100% flock mortality in chickens, production losses, and the enforcement of trade restrictions [3]. Virulent strains have a multi-basic amino acid cleavage site (CS) sequence in the fusion (F) protein with a phenylalanine at position 117 [4, 5]. By comparison, avirulent strains generally have a CS containing fewer basic amino acids and a leucine at position 117, restricted to cleavage by trypsin-like proteases [4, 6]. Avirulent strains of APMV-1, including transmitted vaccine strains, generally cause subclinical infections or mild disease but require pathotyping to understand the risk to the poultry sector as these viruses can adapt following infection of different species. This report adopts the updated classification and nomenclature system of Dimitrov et al. [7], which maintains two APMV-1 classes (I and II) with class II APMV-1 subsequently split into 20 distinct genotypes.

The last reported case of virulent APMV-1/NDV in Great Britain (GB) occurred in the grey partridge (*Perdix perdix*) in 2006 [8]. Detections of avirulent APMV-1 in GB are reported more commonly. In this report, the diagnostic, virological, and epidemiological investigations of three suspected cases of NAD are described where avirulent APMV-1 was detected following negation of notifiable ND. The first case derived from the ‘testing to exclude’ (TTE) scheme, which further highlights the value of the scheme since its introduction in 2014 as a valuable additional early warning surveillance strand for ND that complements the existing statutory surveillance activities for NAD in GB [9, 10]. Through the TTE scheme, veterinarians in clinical practice in GB can request laboratory testing of chicken or turkey flocks where the involvement of avian influenza (AI) or ND is not formally suspected, but neither NAD can be excluded from the differential diagnosis of a flock health or production problem. The primary aim of the scheme is therefore the rapid exclusion of NAD where the clinical presentation does not formally suggest NAD.

## Methods

### Clinical background and submission of samples from the three cases

#### Case 1

The first case occurred in April 2015, when clinical signs of tracheitis, synovitis, and an increase in mortality and haemorrhagic enteritis were reported in two of the four populated houses at a chicken meat production farm in Shropshire. Age of the birds ranged from 38 days to 45 days at the time of the investigation. These were housed intensively indoors with manual ventilation and lighting. However, the biosecurity on the farm was poor, including no wheel wash on entry, with the possibility of indirect fomite transmission of disease into houses. The flock was unvaccinated for ND and infectious bronchitis. *Staphylococcus* and *Enterococcus* were found from culture of carcass tissues following post-mortem examination (PME) by a private veterinary surgeon (PVS). Consistent with TTE requirements (http://ahvla.defra.gov.uk/vet-gateway/tte/nad.htm), the prescribed number of oropharyngeal and cloacal swabs per epidemiological group (birds held, kept, or handled in such a manner that they shared the same likelihood of exposure to APMV-1) (20 of each) were submitted from four houses (house A, house B, house C, and house D) for AI and ND exclusion testing using validated, internationally recognised AIV and APMV-1 real-time reverse transcription polymerase chain reaction (rRT-PCR) methodology [9]. Oropharyngeal and cloacal swabs were pooled as described [11] for influenza A virus and APMV-1 rRT-PCR screening, respectively [12, 13]. Positive results for APMV-1 were obtained from swabs from house A only, leading to an escalation of the TTE case into a statutory NAD investigation. Samples from the remaining three poultry houses were negative.

Although there was a history of clinical disease and mortality peaks in all four houses, at the time of the official veterinary enquiry, mortality and clinical disease had reduced across all four houses, where clinical signs were observed only in house A (containing 7000 birds). Birds in the other three houses were reported as normal following syndromic assessment. Official samples comprising oropharyngeal and cloacal swabs and clotted bloods (20 of each) and seven carcasses were collected from the affected house and submitted for statutory NAD investigation.

#### Case 2

In August 2015, a statutory NAD investigation was conducted in one house of 39,600 birds (house 4) in a chicken broiler production unit in Herefordshire containing a total of approximately 140,000 birds (30 days old). There were four other houses with birds on the site. The affected house had a sudden spiked increase in mortality (>100 birds in a 24-hour period), neurological signs (tremors in heads and legs), and mild diarrhoea. The PVS considered whether botulism or another form of toxicity had been involved. PME findings were unremarkable, except for green-coloured diarrhoea in a single bird, but ND was predominant as differential diagnosis due to the rapid mortality and the neurological signs. No respiratory or digestive signs were present at the time of the visit, reducing the suspicion of AI. Spatial location of the affected or dead birds may have indicated that the infection was spreading outwards from the centre of the house by direct contact and contact with secretions. This area was not close to any evident opening to the outside, but was close to the door used by staff. Infection via fomite (i.e., footwear) was a very early suspicion. Oropharyngeal and cloacal swabs and blood samples from 20 individual birds and six carcasses from the affected house (house 4) and from one other house (house 2; containing 16,000 birds) were submitted for statutory NAD investigation.

#### Case 3

A statutory NAD investigation in October 2015 on premises in Wiltshire containing 575 laying ducks (20 months old), 200 ducklings (7–8 weeks old), and 65 mixed species of free-range poultry. This followed a 40–45% drop in egg production over a three-day period with the ducks showing respiratory signs. Only one duck had died, but PME revealed pathological lesions consistent with upper respiratory tract infection. A larger sample set of 60/60/60 oropharyngeal swabs/cloacal swabs/clotted bloods was submitted for virological investigation from the laying group of ducks along with two carcasses from overnight deaths, plus a 20/20/20 sample set collected from the other free-range birds (three geese, nine ducks, and eight chickens).

### Virus isolation

Virus isolation (VI) in 9- to 11-day-old specific pathogen-free (SPF) embryonated fowls’ eggs (EFE) was performed using the four standard pooled tissue suspensions (pooled tissue homogenates of 10% [w/v] in antibiotics of (i) brain, (ii) lung and trachea, (iii) mixed viscera [heart, kidney, spleen, liver], and (iv) intestines) extracted from each of the carcases from case 1 and case 2, and on pools of up to five oropharyngeal or cloacal swabs submitted from the statutory NAD investigations according to the internationally recognised standard European Union (EU) and World Organisation for Animal Health (WOAH, founded as OIE) methods (WOAH [1]; EU [14]). Preparation of the tissue homogenates for VI was as previously described [15]. The oropharyngeal and cloacal swabs were each added to 1 ml of brain–heart infusion broth (BHIB) containing antibiotics and inoculated into the eggs as described [15]. Allantoic fluids were harvested at two and six days post-inoculation, and samples positive for a haemagglutinating agent were tested for the presence of H1-H16 AIV and APMV-1 haemagglutinating agents by haemagglutination inhibition test (HAIT) subtyping as described (WOAH [1]; EU [14]).

### Polymerase chain reaction

Total nucleic acid was extracted from the single oropharyngeal and cloacal swabs, and four standard tissue suspensions (prepared as described for attempted VI) submitted from the statutory NAD investigations using the QIAmp viral RNA BioRobot kit in conjunction with a Universal BioRobot (QIAGEN, United Kingdom [UK]). Nucleic acids were screened by rRT-PCR for (i) generic detection of influenza A virus targeting the matrix (M) gene [12] and (ii) for specific detection of H5 and H7 AIVs [12, 16]. Samples producing a threshold cycle (C_T_) value <36.0 were considered positive [12, 17]. All samples were simultaneously screened for APMV-1 using a large polymerase (L) gene-specific rRT-PCR [13]. A positive result using this assay was denoted by a C_T_ value <37.0. All PCR amplifications were carried out in an MX3000P qPCR System (Agilent).

### Whole-genome sequencing and phylogenetic analysis

Where VI was successful, whole genome sequence (WGS) data were generated. The extracted RNA was converted to cDNA using the SuperScript IV First-Strand Synthesis System with random hexamers (ThermoFisher), and then to double-stranded cDNA using the NEBNext Ultra II Non-Directional RNA Second Strand Synthesis Module (New England Biolabs). The double-stranded cDNA was then purified and concentrated using Agencourt AMPure XP beads (Beckman Coulter) and incubated at room temperature for 5 minutes and eluted in 10 μL of 1 M Tris–HCl pH 7.5 (Sigma), before quantification using the QuantiFluor dsDNA System (Promega). For preparation of the sequencing library, 1 ng of purified dsDNA was used as the template and the library generated using the Nextera XT kit (Illumina). Sequencing libraries were run on either a MiSeq or NextSeq 550 (Illumina) with 2x150 base paired-end reads. Raw sequencing reads were assembled using a custom script: denovoAssembly.sh (https://github.com/AMPByrne/WGS/blob/master/denovoAssembly_Public.sh (accessed 1 June 2022).

Once the consensus sequence had been generated for the samples, the F protein sequences were combined with appropriate APMV-1 sequences obtained from the NDV consortium sequence database (https://github.com/NDVconsortium/NDV_Sequence_Datasets). Sequences were aligned using Mafft v7.487 [18] and phylogenetic trees inferred using the maximum-likelihood approach in IQ-Tree v2.1.4 [19] with ModelFinder [20] to determine the appropriate phylogenetic model and 1,000 ultrafast bootstraps [21].

### Serology

All sera were separated from the clotted blood samples and tested using the haemagglutination inhibition test (HAIT) to detect H5 and H7 AIV subtype-specific and APMV-1 antibodies according to internationally recognised standard methods [1, 14]. The H5 and H7 AIV antigens were utilised as described previously [22]. Doubling dilutions of sera were mixed with a standard concentration of AIV or APMV-1 antigen, and chicken red blood cells were used to detect the presence of non-inhibited antibodies. HAIT titres equal to or greater than 2^4^ (1/16) were considered positive.

### Intracerebral pathogenicity index (ICPI)

An ICPI test was performed on an egg-amplified APMV-1 isolate from pooled intestine from case 1 and on an isolate from the oropharyngeal swab pool from case 2 (house 2) to determine the virulence of each virus using day-old chicks (*Gallus gallus*) inoculated via the intracerebral route according to the internationally recognised method [1]. No ICPI test was performed for case 3 as no virus was isolated combined with the improving clinical picture, increased feed intake, and egg production on the premises.

## Results

### Case 1

Two of the four cloacal swab pools and one out of four oropharyngeal swab pools from one of the four houses (house A) tested under the TTE scheme were positive by rRT-PCR for APMV-1 nucleic acid, indicating potential NDV infection (Table 1). Subsequent statutory notifiable disease investigation on samples from house A confirmed detection of APMV-1 viral RNA in 25% of oropharyngeal swabs (5/20) and 20% of cloacal swabs (4/20). Overall, 9/20 birds (45%) had an rRT-PCR-positive result from either an oropharyngeal or cloacal swab (Table 1). All four standard pooled tissue samples also yielded rRT-PCR-positive results for APMV-1 RNA. All AIV-specific assays were negative (rRT-PCR and serology). Following two passages in EFE, a haemagglutinating agent was identified in pooled brain, pooled intestines, pooled viscera, and pooled trachea and lungs, and in two of the four cloacal swab pools and in one out of four oropharyngeal swab pools. Infectious APMV-1 was isolated from pooled intestines. HAIT yielded positive anti-APMV-1 titres (≥1/16) in 50% of the birds tested (10/20) and negative titres (1/2–1/8) in 25% of the birds tested (Table 1). Taken together, the rRT-PCR and VI results indicated active infection with APMV-1, with the HAIT results suggesting that some birds were in a convalescent phase of infection. The ICPI test performed on the isolated virus from the pooled intestines demonstrated an avirulent outcome (index of 0.0), therefore negating the presence of NDV. Laboratory results were supported by an improving clinical picture on the premises, and the investigation was closed. Phylogenetic analyses revealed an APMV-1 identical to class II/genotype I.1.1 sequences. These sequences contained an F protein CS motif (GKQGRL) typical of an avirulent APMV-1. The virus (APMV-1/Chicken/United Kingdom/013422/2015) shared 99.94% genetic identity (constituting a single nucleotide change in the F protein sequence) to the Queensland V4 strain (Supplementary Figure S1).

**Table 1.** Summary of the rRT-PCR and HAIT results from the three cases of avirulent APMV-1 in GB

Case no.	Submission type^a^	House	No. birds sampled/tested	rRT-PCR (no. positive/no. tested)	IS^b^	Negative^c^	Positive
1	TTE	House A	20	3/8 pools	n/a^d^	n/a	n/a
		House B	20	0	n/a	n/a	n/a
		House C	20	0	n/a	n/a	n/a
		House D	20	0	n/a	n/a	n/a
	NAD investigation	House A	20	9/20	0	50	50
			7 carcasses^e^	4/4	n/a	n/a	n/a
2	NAD investigation	House 4	20	0	0	100	0
			6 carcasses	0	n/a	n/a	n/a
		House 2	20	17/20	0	85	15
			6 carcasses	2/4	n/a	n/a	n/a
3	NAD investigation	House A	60	5/60	53.3	43.3	3.3
		House B	20	11/20	30.0	30.0	40.0

a Submission type will be either under the “testing to exclude” (TTE) scheme or from a notifiable avian disease (NAD) investigation.

b IS, insufficient volume available to test (percentage of samples shown).

c Negative serology, <1/2, 1/2–1/8; positive serology, ≥1/16. Percentage of samples shown in each case.

d n/a, not applicable. Sera are not submitted for testing under the TTE scheme.

e Four standard tissue pools (brain, lung and trachea, mixed viscera, and intestines) were prepared from the carcasses.

### Case 2

**Figure 1.**
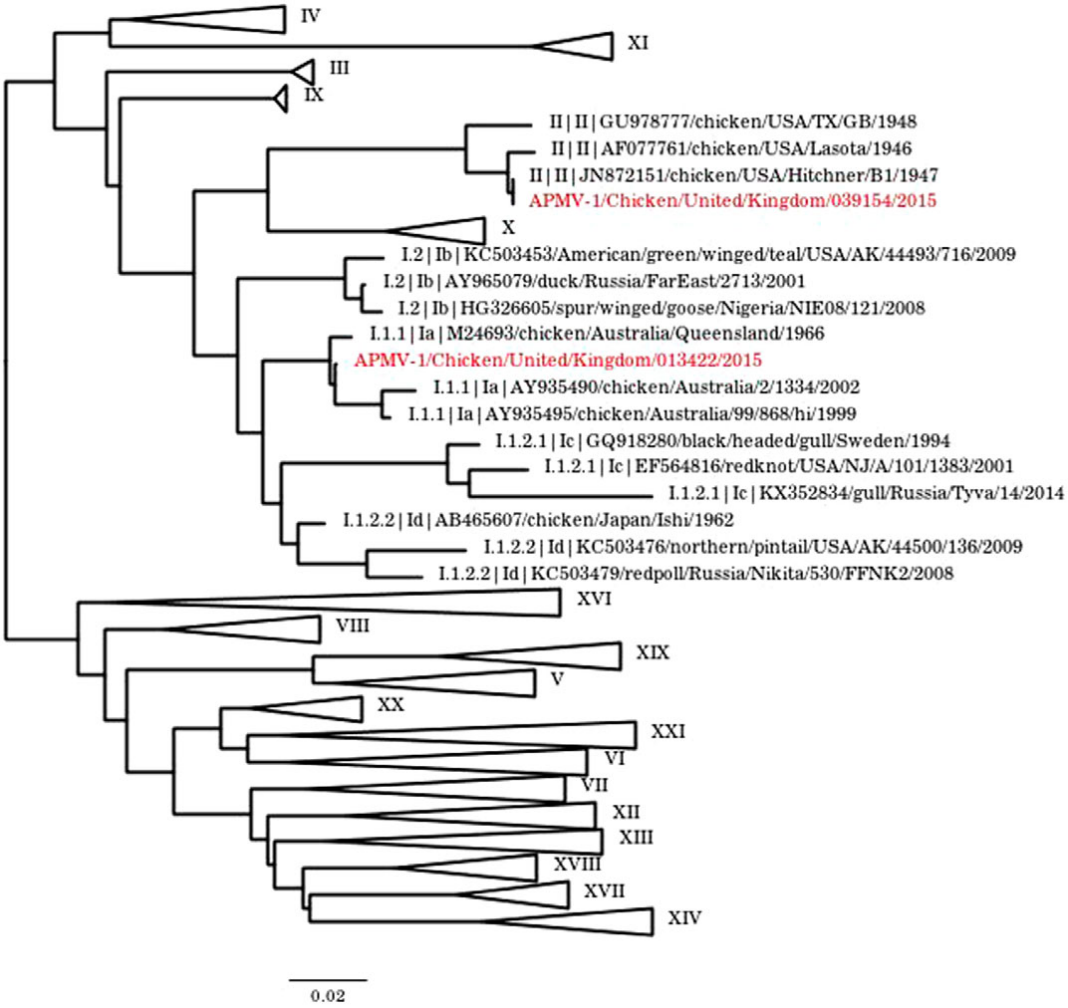
Phylogenetic analysis of case 1 (virus isolate APMV-1/Chicken/United Kingdom/ 013422/2015 from submission AV526-15; Table 2) and case 2 (virus isolate APMV-1/ Chicken/ United Kingdom/039154/2015 from submission AV1106-15; Table 2) based on F protein ORF of different class II APMV-1. Sequences were aligned in MAFFT, and phylogenetic tree constructed using the Maximum-Likelihood method and 1000 bootstraps. F protein sequences were obtained from NDV consortium sequence database. The two sequences are highlighted in red.

Laboratory investigation confirmed APMV-1 infection in both houses (Table 1). Differential diagnoses of AI (rRT-PCR and serology) were negative. Swabs and carcasses submitted from house 4 were negative for APMV-1 by rRT-PCR, and no haemagglutinating viruses were isolated. However, even though all individuals demonstrated negative serological titres against APMV-1 (1/2–1/8), the weak titres suggested previous exposure to an APMV-1 strain with low virulence, vaccination stress, or previous vaccination history of the birds in this house. In comparison, oropharyngeal swabs from house 2 were rRT-PCR-positive for APMV-1 RNA in 85% of birds (17/20), with positive cloacal swabs in only a single bird (1/20) (Table 1). APMV-1 RNA was also detected in pooled carcass tissues from house 2 (pooled brain, and pooled lung and trachea). Virus isolation yielded a haemagglutinating agent in pooled swabs and pooled lung and trachea, and APMV-1 was isolated from pooled lung and trachea and from one oropharyngeal swab pool. HAIT results demonstrated positive anti-APMV-1 titres in 15% of birds in house 2 (3/20), whilst 85% of birds exhibited negative titres (17/20) (Table 1), indicative of an active infection with APMV-1, and that some birds were convalescing. The ICPI test score (0.2) on the isolated virus from the oropharyngeal swab pool was less than 0.7 and ruled out the presence of NDV on the premises. Phylogenetic analysis on the RNA extracted on the virus isolated from the oropharyngeal swab pool (APMV-1/Chicken/United Kingdom/039154/2015) confirmed an avirulent class II/genotype II APMV-1. The sequences contained an F protein CS motif (GRQGRL) typical of an avirulent APMV-1 isolate (Figure 1 and Supplementary Figure S1).

**Table 2.** Summary of the detections of avirulent APMV-1 in GB since 2005, including the affected species, virus genotype, F protein cleavage site sequence, and ICPI

Year	APHA submission reference	Species	Genotype	Cleavage site sequence	ICPI^a^
2005	AV868–05	Pheasant	1 (I)	GKQGRL	0.00
2006	AV7739–06	Turkey	2 (II)	GRQGRL	0.025
2007	AV955–07	Chicken	1 (I)	GKQGRL	0.0375
2008	AV2262–08	Turkey	1 (I)	GKQGRL	nd^b^
2009	AV1328–09	Turkey	2 (II)	GRQGRL	0.29
	AV1336–09	Chicken	2 (II)	GRQGRL	0.69
2010	AV291–10	Chicken	1 (I)	GKQGRL	0.00
	AV776–10	Duck	1 (I)	GKQGRL	0.00
2015	AV526–15	Chicken	1 (I)	GKQGRL	0.0
	AV1106–15	Chicken	2 (II)	GRQGRL	0.2
	AV1382–15	Duck	1 (I)	GKQGRL	nd

a ICPI, intracerebral pathogenicity index.

b nd, not done.

### Case 3

Table 1 summarises the outcomes of the virological investigations from the third case. Tests for differential diagnosis of AIV (rRT-PCR and serology) were all negative. However, infection with APMV-1 was confirmed through detection of viral RNA in 8.3% of cloacal swabs (5/60) from house A (Table 1). All oropharyngeal swabs were negative. The carcasses from house A were not tested. HAIT results demonstrated positive anti-APMV-1 titres in 3.3% of birds (2/60), whilst 43.3% (26/60) of birds had negative titres (Table 1). However, 53.3% of the blood samples (32/60) from this house could not be tested due to insufficient volumes of serum available for testing. The level of seroconversion was higher in the birds from house B where 40% (8/20) demonstrated positive anti-APMV-1 titres. Attempts to isolate virus from either the oropharyngeal or cloacal swab pools was unsuccessful and so the ICPI test could not be undertaken. Overall, the results indicated that the ducks had been actively infected with APMV-1 and that some birds had been convalescing. Due to limited sequencing data, full phylogenetic analysis could not be performed, however, the sequence closely resembled a class II/genotype I.2 APMV-1 virus isolated from wild *Anseriformes* (data not shown). It was determined that this isolate had an avirulent CS motif (GKQGRL), matching closely with other contemporaneous APMV-1 viruses.

## Discussion

Newcastle disease is defined in the WOAH Terrestrial Animal Health Code as an infection of poultry caused by an APMV-1 that meets one of the following criteria for virulence: an ICPI of 0.7 or greater in one-day-old chicks (*G. gallus*), or a sequence of specific amino acid residues in the F protein that have been shown to be associated with virulent strains [1]. However, avirulent strains of APMV-1 are ubiquitous worldwide and continue to be detected occasionally in GB (Table 2). This report describes the detection of three cases of avirulent APMV-1 in GB, and these reactive case studies demonstrated the importance of rapid and sensitive diagnostics that can quickly differentiate avirulent APMV-1 from NDV, thereby enabling safe and early lifting of precautionary disease restriction measures on affected farms.

Of the three cases, two were genetically similar to strains found in wild bird and poultry cases previously detected in Denmark [23], suggesting widespread dispersal and maintenance of viruses via the movement of wild birds. Despite the relatively low numbers of cases detected in GB, the clinical signs observed from infections with avirulent strains of APMV-1 represent a low, but not insignificant, economic impact to poultry farmers due to the restrictions placed on the affected premises until virulent APMV-1 infection (resulting in ND) can be negated, along with the increased morbidity and mortality, and decrease in egg production and/or quality.

Class II/genotype I APMV-1 isolates generally comprise ‘Ulster 2C-like’ (I.1.2) and ‘Queensland V4-like’ (I.1.1) viruses. Both strains generally induce only mild clinical signs in poultry and are used extensively to produce live ND vaccines worldwide. A class II/ genotype I.1.1 APMV-1 was detected in case 1. This virus was genetically 99.94% identical to the Queensland V4 strain of APMV-1. Between 2005 and 2015, class II/genotype I APMV-1 was isolated from eight poultry premises in GB (Table 2). All premises reported clinical signs in chickens ranging from mild respiratory distress, diarrhoea and egg drop to mortalities. The vaccination history of all flocks was not complete, but some were vaccinated against NDV using commercially available vaccines. Importantly, in GB, the most common licensed vaccines that are based on class II/genotype I viruses are derived from the ‘Ulster 2C’ strain, thereby likely excluding vaccine as the source of infection in case 1. Avirulent class II/genotype I viruses are commonly associated with wild birds and therefore may be a possible source of infection [24]. The class II/genotype I.2 virus detected in case 3 matched closely with other APMV-1 strains isolated from wild *Anseriformes*, suggesting that this is a stable genotype that can readily transmit to and infect domestic waterfowl and ducks. Class II/genotype II APMV-1 was detected in the second case and was found to be genetically similar to several viruses used as vaccine strains. Given that ‘Queensland V4-like’ (I.1.1) virus itself is not found in wild waterfowl, we conclude that cases 1 and 2 most likely relate to unintentional infection with live vaccine strains (Figure 1). The wild-type class II/genotype II viruses had their origins in North America and have varying impacts ranging from the virulent ‘Texas GB/48’ strain to the avirulent ‘B1/47’ strain, which induces only mild respiratory signs in chickens [25]. Included in this group of viruses is the lentogenic ‘LaSota’ strain, which along with ‘B1/47’ is commonly used in live vaccine preparations worldwide, including a number licensed vaccines for use in GB. The analysis of vaccine strains isolated from wild birds in Egypt suggests that the extensive use of live attenuated vaccines in the poultry industry may lead to the transmission of vaccine strains of mild virulence from vaccinated poultry to wild birds or naïve poultry [26].

Although avirulent strains of APMV-1 cause limited or no clinical disease with severity generally only seen in multifactorial cases, rapid diagnosis remains essential as these pathogens still constitute a small risk to poultry populations. The zoonotic potential of APMV-1 is thought to be low, with infections in humans causing a self-limiting disease presenting as conjunctivitis (pink eye), laryngitis, and mild flu-like symptoms [27, 28]. Following incursion of an avirulent APMV-1 into a susceptible poultry population, sustained transmission and circulation within that population can potentially lead to mutation of the virus to virulent forms, which may lead to disease in susceptible populations [2, 23, 29, 30]. As few as ten serial passages of an avirulent strain in chickens can cause a series of sequential changes at the F protein CS of three point mutations, which results in development of a virulent phenotype [31]. The isolation of genotype 1 ‘Queensland V4-like’ APMV-1 in GB poultry occurred in 2005 from chickens on a farm in the west of England (unpublished). More recently, deep sequencing of a virulent Malaysian APMV-1 isolate identified a natural recombination event between class II/ genotype II and a class II/genotype VII virus, highlighting the propensity of RNA viruses, such as APMV-1, to evolve [32]. Rapid detection of these viruses is therefore imperative. In this regard, and as evidenced by the first case reported here, the TTE scheme facilitates the early detection of potential incursions following syndromic suspicion of disease. This study also highlights the need for careful use of vaccines, since vaccines are considered one of the biggest drivers of APMV-1 diversity. Enhanced differentiation between circulating wild-type viruses and vaccine strains through the deployment of improved tools for vaccination and detection of APMV-1 would benefit the poultry sector both within GB and globally.

## Supporting information

Reid et al. supplementary material Reid et al. supplementary material

## Data Availability

Data will be supplied upon request.

## Supplementary material

For supplementary material accompanying this paper visit http://doi.org/10.1017/S0950268823001255. click here to view supplementary material
