# Supplementary figures and images for: Understanding the disease and economic impact of avirulent avian paramyxovirus type 1 (APMV-1) infection in Great Britain
Source: Epidemiol Infect. 2023 Aug 25;151:e163. doi: 10.1017/S0950268823001255 (PMC10600730; doi:10.1017/S0950268823001255)

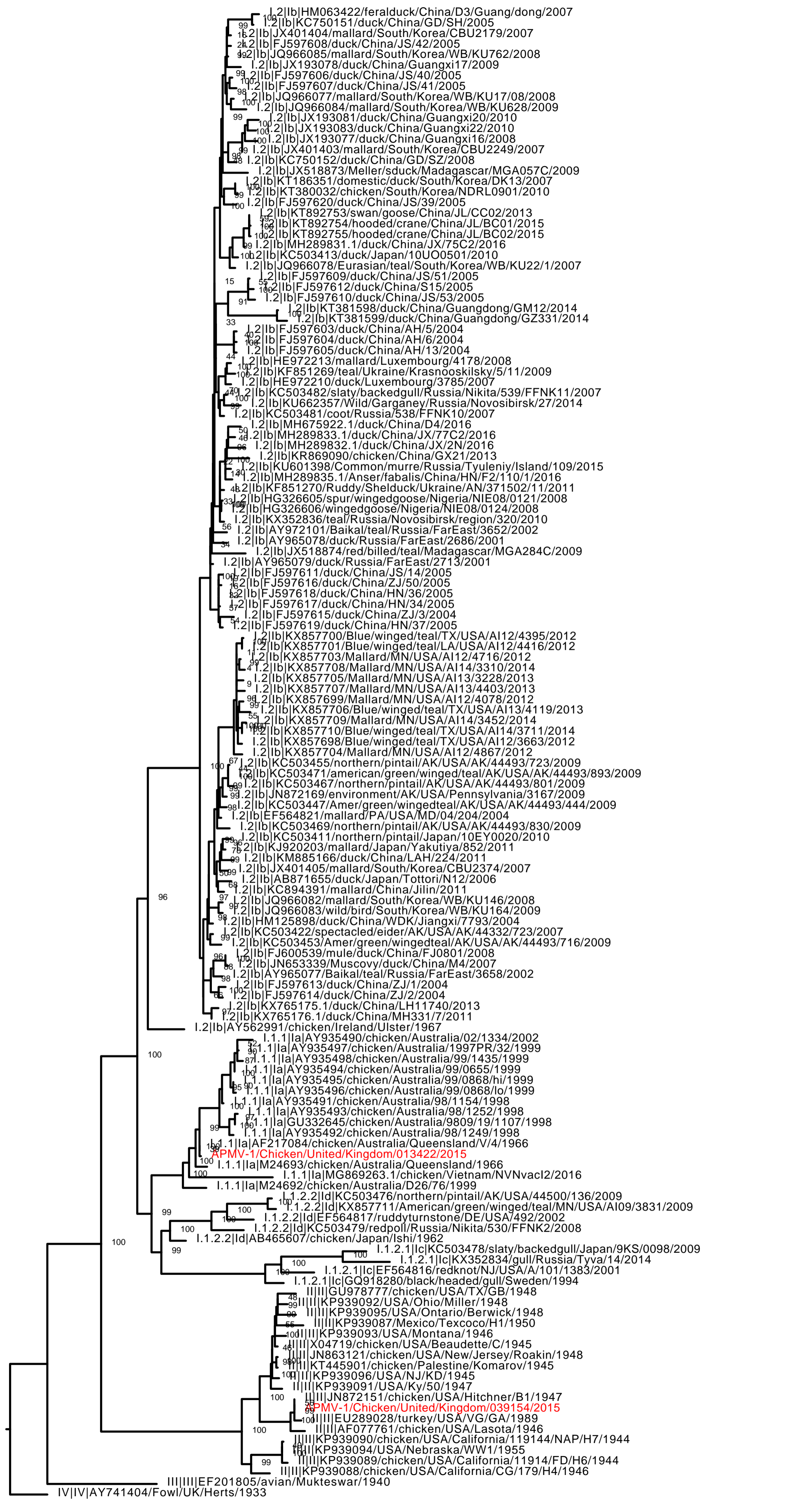

Supplement: Reid et al. supplementary material [file S0950268823001255sup001.pdf]
